# HPV18 L1 and long control region sequences variation and E6/E7 differential expression in nasopharyngeal and cervical cancers: a comparative study

**DOI:** 10.1186/s13027-023-00560-5

**Published:** 2023-12-01

**Authors:** Sheila Santa, Charles A. Brown, Patrick K. Akakpo, Lawrence Edusei, Osbourne Quaye, Emmanuel A. Tagoe

**Affiliations:** 1 Department of Biochemistry, Cell and Molecular Biology, West African Centre for Cell Biology of Infectious Pathogens (WACCBIP), University of Ghana, Accra, Ghana; 2 Department of Medical Laboratory Sciences, University of Ghana, Accra, Ghana; 3 Pathology Department, Medical School, University of Ghana, Accra, Ghana; 4 Pathology Department, School of Medical Sciences, University of Cape Coast, Cape Coast, Ghana; 5 Pathology Without Borders, Laterbiokorshie, Accra, Ghana

**Keywords:** Human papillomavirus, Long control region, L1 gene, E6, E7, Cervical cancer, Nasopharyngeal cancer

## Abstract

**Background:**

The role of high-risk human papillomaviruses (hr-HPVs) in cervical cancer (CC) pathogenesis has long been established. Knowledge about the involvement of hr-HPVs in the etiology of nasopharyngeal cancers (NPC) was not well appreciated until the early 2000s when a clear link began to emerge. However, it is not clear whether HPV oncogenesis in the different epithelial cancers is associated with L1 gene and long-control region (LCR) sequences variation. This study aimed to investigate the HPV18 L1 gene and LCR sequences variation in cervical and nasopharyngeal biopsies, and assessed E6 and E7 genes expression level in both cancers.

**Method:**

Four-hundred and three (403) formalin-fixed paraffin-embedded tissues originating from nasopharyngeal (NPC) (279) and cervical (CC) (124) sites were collected from a pathology laboratory, Pathologist Without Borders, Accra, Ghana. Haematoxylin and eosin staining was carried out to confirm the presence of cancer on prepared biopsy sections. DNA was extracted from the confirmed cancer biopsies, followed by PCR using MY09/GP5+ /6+ primers to detect the presence of HPV and specific primers for the amplification of L1 gene and LCR. Sanger sequencing was carried out to determine HPV genotypes, and L1 and LCR sequences variant of HPV18s in CC and NPC biopsies. The HPV18 E6/E7 mRNA expression pattern in both cancers was determined using RT-qPCR.

**Results:**

Most of the NPC (45%) and CC (55%) biopsies were HPV18 positive. Comparison of HPV18 L1 sequences obtained from cervical and nasopharyngeal cancer tissues, the L1 sequences from the NPC were highly dissimilar with a 59–100% variation among themselves, and in relation to the reference strains. However, the L1 sequences from the CC were more similar with a 91.0–100% variation among the amplified sequences. Also, the LCR sequences from CC were quite different relative to that of NPC. Results for the differential expression of E6/E7 in the two cancers showed a higher fold change in E6 expression in the CC tissues than the NPC tissues while a reverse expression pattern was found for E7 gene.

**Conclusion:**

The current study reports for the first-time variations in HPV18 L1 and LCR sequences, and differential expression of E6/E7 genes in NPC compared to CC, suggesting a possible adaptation mechanism of the virus at different cancer sites.

**Supplementary Information:**

The online version contains supplementary material available at 10.1186/s13027-023-00560-5.

## Introduction

Cervical and nasopharyngeal cancers are two well-known pathogen-associated cancers [1]. Cervical cancer (CC) is the fourth leading cause of death globally in women, accounting for an estimated 604 000 new cases in 2020 with about 90% of these cases occurring in low- and middle-income countries [2]. The link between CC and human papillomavirus (HPV) infection has been established with an estimated 99% of CC being attributed to HPV infection [3, 4]. Though nasopharyngeal cancer (NPC) is not a very common cancer globally, an estimated 129,079 new cases were diagnosed worldwide in 2018 and approximately 72,987 NPC-related deaths occurred within the same period [5, 6]. The major risk factors for NPC have been Epstein-Barr virus and tobacco smoking. In recent times, however, HPV infection has been implicated in the outcome of NPC [5, 7, 8].

Generally, HPVs are small, non-enveloped, icosahedral deoxyribonucleic acid (DNA) viruses associated with mucosal and cutaneous epithelial infection [9]. The genomes of HPVs comprise approximately 8000 base pairs, with about 8 open reading frames (ORFs) transcribed from a single DNA strand. The ORF are divided into three main parts namely the early (E) region that encodes proteins (E1–E7), the late (L) region encodes the structural proteins (L1–L2) and a non-coding part called the long control region (LCR) [10]. The E1 and E2 proteins are factors involved in recognition of the origin of replication, E4 proteins are involved in the late stages of the viral life cycle and E5 is thought to function during both early and late phases [11]. The E6 and E7 proteins are the main oncoproteins of the virus and are known to target a number of anti-proliferative regulators of the cell cycle, especially p105Rb and p53 [12]. The E6 oncoproteins principally promote the ubiquitin-dependent proteasome degradation of p53 in HPV-infected cells, while the E7 oncoprotein mainly targets the inactivation of pRb and the downregulation of E2F [13]. The L1 and L2 proteins are involved in the assembly of viral capsomers, which form icosahedral capsids around the viral genome hence involved with immune evasion [14]. The long control region (LCR), also known as the upstream regulatory region (URR) is a non-coding region which has binding sites for several transcription and regulatory factors necessary for the replication and transcription of viral DNA including the E6 and E7 oncogenes [15]. HPVs have been shown to have preference for specific anatomical sites and site-specific infection is characterized with distinctive clinical pathologies [16]. A Zimbabwean study which sequenced a segment of the HPV L1 gene of 35 different HPV types from the anal and vaginal sites of 144 women revealed tens of thousands of genetic variants, suggesting intra-host variability [17]. Thus, tissue tropisms and host immune status could contribute to rates of HPV diversity [16, 17].

Several high risk HPV (hr-HPV) types (16, 18, 31, 33, 35, 39, 45, 51, 52, 56, 58, 59, 66, and 68) have been associated with cervical cancers, and HPV 16 and 18 are known to account for about 70% of these cancers [12]. Low risk HPV types (6, 11, 42, 43, and 44) are implicated in genital, oral, and throat warts [18]. Approximately 30 anogenital HPV types have been identified and reported to spread through sexual contact, infecting mostly the anogenital regions such as; cervix, vagina, vulva, penis, and anus [19]. HPV 16 and 18 have been implicated in the pathogenesis of NPCs [20–22], and these HPVs are believed to be transmitted orally, mostly through contact of the oral region with the anogenital region such as vaginal, anal, penile and vulva [23]. The strategies that these hr-HPVs employ to persist in the cervical and nasopharyngeal sites have not been clearly investigated. The purpose of this study, therefore, was to investigate the site-specific patterns of HPV18 L1 and LCR sequence variation and E6 and E7 gene expression pattern in nasopharyngeal and cervical cancers.

## Materials and methods

### Study design and population

A retrospective cross-sectional study design was employed. Sample collection and pathological analysis were carried out at Pathologist Without Borders, Accra, Ghana, a private anatomical pathology diagnostics company that reports averagely, 12,000 cases a year. Archived formalin fixed paraffin embedded (FFPE) tissue blocks of cervical lesions and nasopharyngeal lesions diagnosed between January 2019 and December 2021 were retrieved from the tissue bank. In all, 403 FFPE tissues blocks of cervical and nasopharyngeal biopsies were retrieved.

### Ethical approval

Ethical approval to conduct the study was obtained from the Ethics Committee of Basic and Applied Sciences (ECBAS), University of Ghana, with identification number ECBAS 045/21-22.

### Processing of biopsies

The selected FFPE tissue blocks were sectioned using a Leica microtome RM2235 and Leica microtome blade (Biosystems, USA). Ten (10) µm thick sections of seven series were taken from each FFPE tissue using a new blade to avoid cross-contamination.

The sections were transferred into Eppendorf tubes with the blade that was used to section that block. Two sections were fixed on slide and stained with hematoxylin and eosin, and examined under a microscope by a Board-Certified Pathologist to confirm the presence or absence of cancer. Confirmed cervical (51) and nasopharyngeal (48) cancer biopsies, and cervical and nasopharyngeal non-cancer tissues were selected as controls.

### Detection of HPV in cervical and nasopharyngeal FFPE tissues

#### DNA and RNA extraction

Genomic DNA and total RNA were extracted from the cervical and nasopharyngeal cancer tissues using the Quick-DNA/RNA™ FFPE Kits from Zymo-Research (Cambridge, UK). Briefly, five series of 10 µm sections from each FFPE tissue block were deparaffinized using a deparaffinization solution followed by digestion with proteinase K. The lysate was incubated at 55 °C for 4 h and subsequently at 94 °C for 20 min to de-crosslink the sample, and then purified using Zymo-Spin™ column technology according to the manufacturer’s instruction. The flow-through was collected for RNA extraction. The DNA was then eluted in 50 µl of DNA/RNase free water and stored at − 20 °C until use. For the RNA purification, 1 volume of ethanol (95–100%) was added to the flow-through (1:1) and mixed thoroughly. The flow-through was then transferred into a new Zymo-Spin™ IICR column in a collection tube and centrifuged. The RNA remained in the spin column and was eluted in 50 µl of DNA/RNase free water after several steps of purification, according to the manufacturer’s instruction. NanoDrop™ (ThermoFischer, USA) was used to determine the concentration and purity of the DNA and RNA extracted. Extracted DNA and RNA from HPV-infected Hela cells were used as positive controls for the PCRs.

#### HPV detection

Nested-PCR assay was performed using the primers and protocol previously described with some modifications [24]. The first round PCR reaction for detection was carried out in a total volume of 25 µL containing template DNA (2 µL), 12.5 µL enhanced fidelity polymerase OneTaq® Quick-Load® 2X Master Mix with standard buffer (New England Biolabs, USA), 0.2 µM each of MY09/11 primers; forward: 5′-CGT CCM ARR GGA WAC TGA TC-3′ and reverse: 5′-GCM CAG GGW CAT AAY AAT GG-3′ and topped up with deionized water. Amplification was performed in a Techne Prime thermal cycler (Cole Parmer, USA) with the following conditions: 94 °C for 30 s, 49.5 °C for 1 min, and 68 °C for 1 min for a total of 35 amplification cycles. The first cycle was preceded by a 30 s denaturation step at 94 °C. The last cycle was followed by an additional 5 min elongation step at 68 °C. The second round PCR was carried out using 2 µL of the MY09/11 primer PCR product as a template and GP5+ /6+ primer with sequences; forward: 5′-CGT CCM ARR GGA WAC TGA TC-3′ and reverse: 5′-GCM CAG GGW CAT AAY AAT GG-3′.

The same reagents, concentrations and amplification condition were used as for the first round. The MY09/11 primer set amplified approximately a 450 bp fragment within the HPV L1 structural gene while the GP5+ /6+ primer amplified approximately a 140 bp fragment within the 450 bp fragment. The pairing of MY09/11 and GP5+ /6+ primers further amplified a 190 bp fragment from the 450 bp fragment [24].

##### HPV18 LCR amplification

A nested-PCR assay was performed according to the protocol previously described by Awua et al., with some modifications [25].

Primers used for the PCR were as follows; HPV18LCR primer for first round PCR; forward: 5′-GTGTTTGTGGTATGGGTGTT-3′ and reverse: 5′-GTATAGTATGTGCTGCCCAA-3′ and HPV18LCR2 second round PCR; forward: 5′-CGGTTGCATAAACTATGTAT-3′ and reverse: 5′-TCGGTTGCCTTTGGCTTATG-3′.

Briefly, the first round PCR reaction was carried out in a total volume of 25 µL containing template DNA (2 µL), 12.5 µL enhanced fidelity polymerase, OneTaq® Quick-Load® 2X Master Mix with standard buffer (New England Biolabs, USA), 0.2 µM each of primer set and topped up with deionized water.

Amplification was performed in a Techne Prime thermal cycler (Cole Parmer, USA) with the following conditions: 95 °C for 1 min, 57.1 °C for 1 min, 30 secs; and 68 °C for 1 min, 30 secs for a total of 35 amplification cycles. The first cycle was preceded by a 5 min denaturation step at 95 °C. The last cycle was followed by an additional 5 min elongation step at 72 °C. The second round PCR was carried out using 2 µL of the first round PCR product as template. The same reagents, concentrations and amplification condition were used as described above.

##### Genotyping of HPVs by Sanger sequencing

HPV L1 and LCR amplicons from the GP5 + /6 + and HPV18LCRR2 primers were selected for Sanger sequencing after the PCR products have been separated in 2% ethidium bromide-stained agarose gels. The representative samples for sequencing were a single band with molecular weights of 140 bp (GP5 + /6 +) for the L1 gene and 375 bp (HPV18LCRR2) for the LCR. Sample tubes containing the selected amplicons were clearly labelled and shipped for sequencing with instructions.

##### HPV L1 and LCR sequences analysis

Following the sequencing of HPV L1 gene and LCR, the L1 sequences obtained were aligned with HPV18 L1 reference sequences; DQ059013 (Mauritius), EF202155 (African), MH028425 (Italy), EF202147 (European), AY262282 (Asia), and MN689568 (Iran) retrieved from NCBI GenBank Database (https://www.ncbi.nlm.nih.gov/). All retrieved sequences were trimmed along with the sample sequences. Multiple sequence alignment of L1 genes and LCRs nucleotide sequences were separately performed using ClustalX, version 2.0 (European Bioinformatics Institute) [26]. Similarity and percent identity matrix of L1 sequences were analyzed using, BioEdit (version 5.0.9) software programme [27]. Phylogenetic trees using ClustalX Neighbor-Joining (NJ) algorithm, were generated separately for L1 and LCR sequences. The reliability was assessed by the calculation of bootstrap with 1000 replicates.

#### RT-PCR for HPV18 E6 and E7 expression

Ten (10) blocks each of HPV 18 positive cervical and nasopharyngeal cancer tissues, and 10 blocks each of HPV 18 positive cervical and nasopharyngeal non-cancer tissues (controls) were selected to determine E6/E7 mRNA expression. Briefly, 2 μl of total RNA was added to the master mix containing; OneTaq One-Step reaction mix, OneTaq One-Step enzyme mix (25X) (OneTaq® One-Step RT-PCR Kit, New England Biolabs, USA), HPV E6, forward primer: 5′-GTATGGAACAACATTAGAACAGCAA-3′; reverse primer: 5′-GTGGCTTTTGACAGTTAATACACC-3′ and E7, forward primer: 5′-GCATGGAGATACACCTACATTG-3′;

reverse primer: 5’-TGGTTTCTGAGAACAGATGG-3’ in a total volume of 10 µl. Amplification was done using QuantStudio 3 system (ThermoFischer, USA), according to the dye method. The following amplification conditions were used; 55 °C for 10 min, 95 °C for 1 min, 95 °C for 15 s, 60 °C for 1 min, 68 °C for 1 min, and 68 °C for 5 min according to the protocol of OneTaq® One-Step RT-PCR Kit. All reagent reconstitutions were carried out on ice. GAPDH was used as an internal control and the master mix without template RNA was also used as a negative control.

#### Statistical analysis

Statistical analysis was performed with GraphPad prism software version 9.0. Data normality was checked by Shapiro–Wilk test. Normally distributed data was presented as mean and standard deviation whereas non-normally distributed data was depicted as median with interquartile range. Statistical comparisons among and between more than two groups were determined by One-way analysis of variance (ANOVA) or Kruskal–Wallis test. Differences between two groups was analyzed by the two-tailed unpaired t-test or Wilcoxon-sum signed rank test. Associations between categorical variables was determined using Chi square (χ^2^) or Fisher’s exact test. Sequencing data for groups was analyzed using bioinformatic approaches. The fold change in HPV E6 or E7 expression was calculated using the 2^−ΔΔct.^ method. *p*-value < 0.05 was considered statistically significant.

## Results

### Age, sex and pathological classification of cancer tissues

**Fig. 1:**
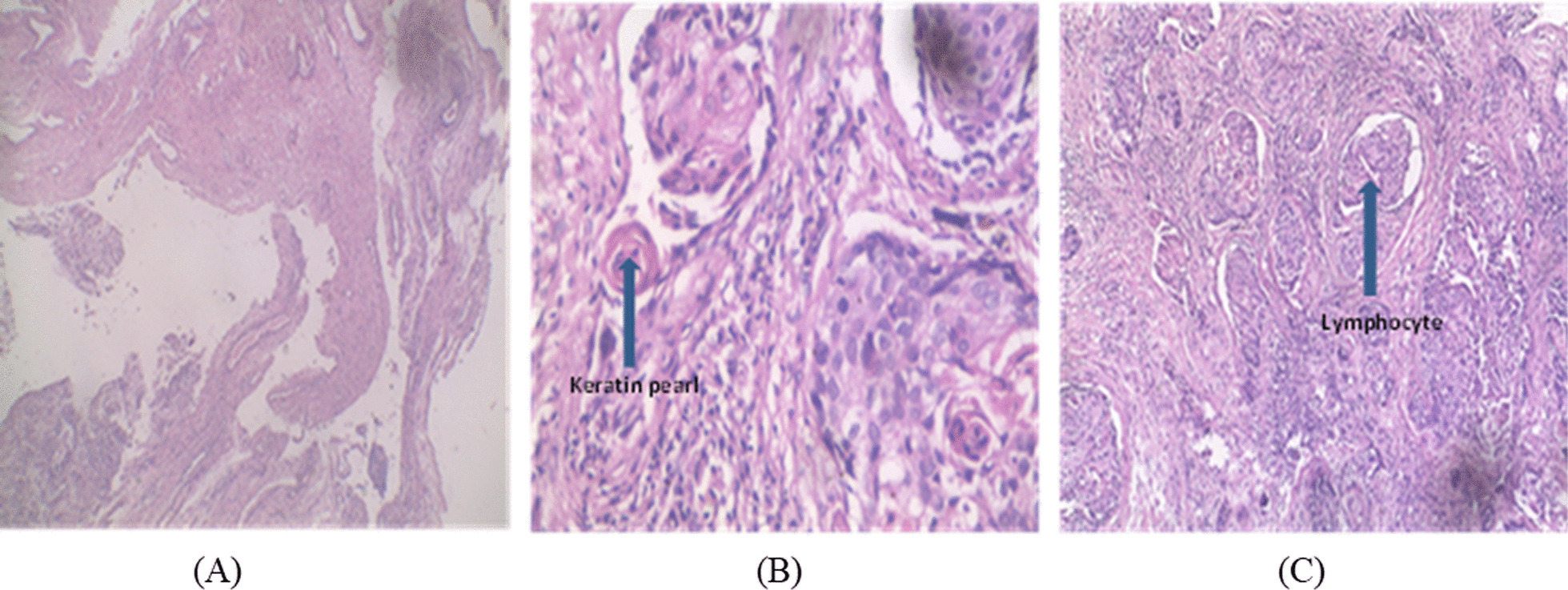
Haematoxylin and eosin staining of epithelial tissues. (**A**) normal (**B**) cancer with keratin pearls (**C**) cancer with infiltrating lymphocytes

Out of the total blocks, 279(69%) were nasopharyngeal and 124(31%) were cervical tissues. Distribution of age and sex of patients from whom the FFPE tissues were taken is presented in Table 1. The median age for patients diagnosed with nasopharyngeal and cervical cancers were 60.5 and 47 years, respectively. Nasopharyngeal cases were overrepresented among men (77.0%) than females. Haematoxylin and eosin staining confirmed 48(17%) nasopharyngeal and 51(41%) cervical squamous cell carcinomas. The predominant immune cell type in the cancer tissues was lymphocytes. Most of the cervical cancers were keratinizing. The undifferentiated nasopharyngeal carcinomas were however, non-keratinizing (Fig. 1). Histopathological classification of the cancers is shown in Table 2.

**Table 1:** Age and sex distribution of patients

FFPE tissues	No. of blocks Retrieved	Age Median (IQR) yrs	No. males	No. female
Total number of cases retrieved	403 (100%)	47 (36–61)	228 (57%)	175 (43%)
Nasopharyngeal tissues	279 (69%)	49 (39–62)	228(82%)	51 (18%)
Cervical tissues	124 (31%)	47 (36–60)	–	124 (31%)
Nasopharyngeal cancer	48 (17%)	51 (36–63)	37(77%)	11 (23%)
Cervical cancer	51 (41%)	44 (35–55)	–	51 (100%)

*IQR* interquartile range

**Table 2:** Histopathological classification of cancers

	Poorly differentiated Carcinoma,	Undifferentiated Carcinoma	Moderately differentiated Carcinoma	Well differentiated Carcinoma
Nasopharyngeal	10 (21%)	14 (29%)	18 (37.5%)	6 (12.5%)
Cervical	18 (35%)	0	15 (30%)	18 (35%)

### Detection and distribution of HPV in cervical and nasopharyngeal cancer tissues

**Fig. 2:**
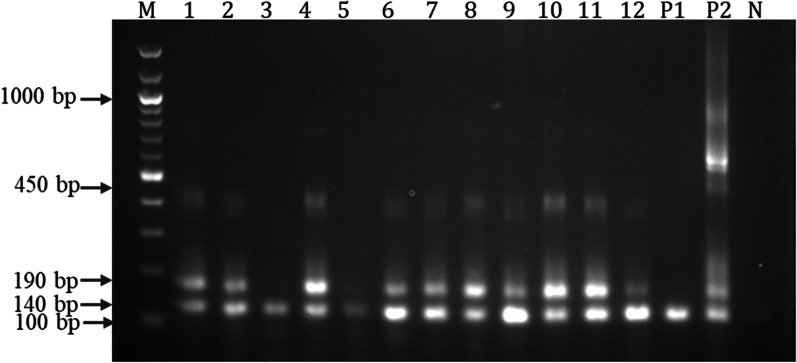
Detection of HPV cervical and nasopharyngeal biopsies using MYO9/GP5 + /6 +  (MY/GP). Representative ethidium bromide-stained 2.5% agarose gel electropherogram of PCR for the identification of HPV L1 region. M = 100 bp molecular ladder; HPV positive samples = 1–12; P1 = positive control for first round PCR; P2 = positive control for nested PCR. The MY primer set amplified a 450 bp fragment within the HPV L1 region, the GP primers amplified a 140 bp fragment within the MY amplified region and the pairing of MY and GP primers further amplified a 190 bp fragment from the 450 bp region. N = negative control

Out of the 99 cancer tissues (51 cervical and 48 nasopharyngeal) used for the study, 84(84.5%) were HPV positive. Forty-seven (47) out of the 51 cervical cancer tissues were HPV positive representing 92% of the cervical tissues while 37 out of the 48 nasopharyngeal cancer tissues were HPV positive representing 77% of nasopharyngeal cancer tissues. HPV infection was significantly associated with cervical cancer (*p* < 0.01) (Table 3). A representative gel image for HPV detection is shown in Fig. 2.

**Table 3:** Association of HPV with cancers

Cancers	HPV^+^ n (%)	HPV^−^ n (%)	Chi square (χ^2^)	p-value
Cervical (N = 51)	47 (92.2)	4 (7.8)	8.589	0.0033*
Nasopharyngeal (N = 48)	37 (77.1%)	11 (22.9)		

*p-value less than < 0.05 was considered statistically significant

### Sequence and phylogenetic analysis of HPV18 L1 genes

**Fig. 3:**
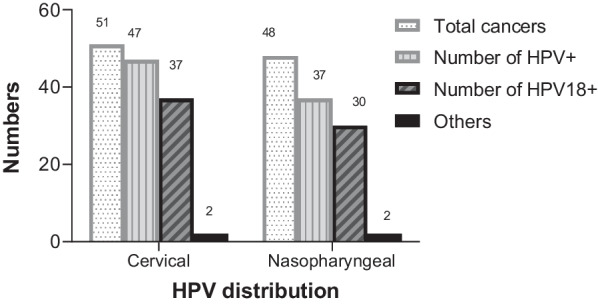
Distribution of HPV in cervical and nasopharyngeal cancer tissues

From the total of 84 HPVs detected in cervical and nasopharyngeal tissues, 71 HPV L1 genes were successfully sequenced. HPV18^+^ were 67(94.4%) made up of 37 (55.2%) cervical and 30 (47.8%) nasopharyngeal cancer biopsies. Other 4 HPV types (5.6%) were HPV16 1(25.0%) and HPV6 3(75.0%). One HPV6 and HPV16 were found in cervical and 2 HPV6 were seen in the nasopharyngeal cancer tissues (Fig. 3).

#### HPV 18 L1 sequence comparison

HPV18 L1 sequences detected in cervical cancer were highly identical, with percentage nucleotide identity ranging from 91.0 to 100%. Contrastingly, HPV18 L1 sequences detected in nasopharyngeal cancers varied with percentage nucleotide identities ranging from as low as 59–100% (Additional file 1: Table S1 and Additional file 2: Table S2).

#### Phylogenetic analysis of HPV 18 L1 gene

**Fig. 4:**
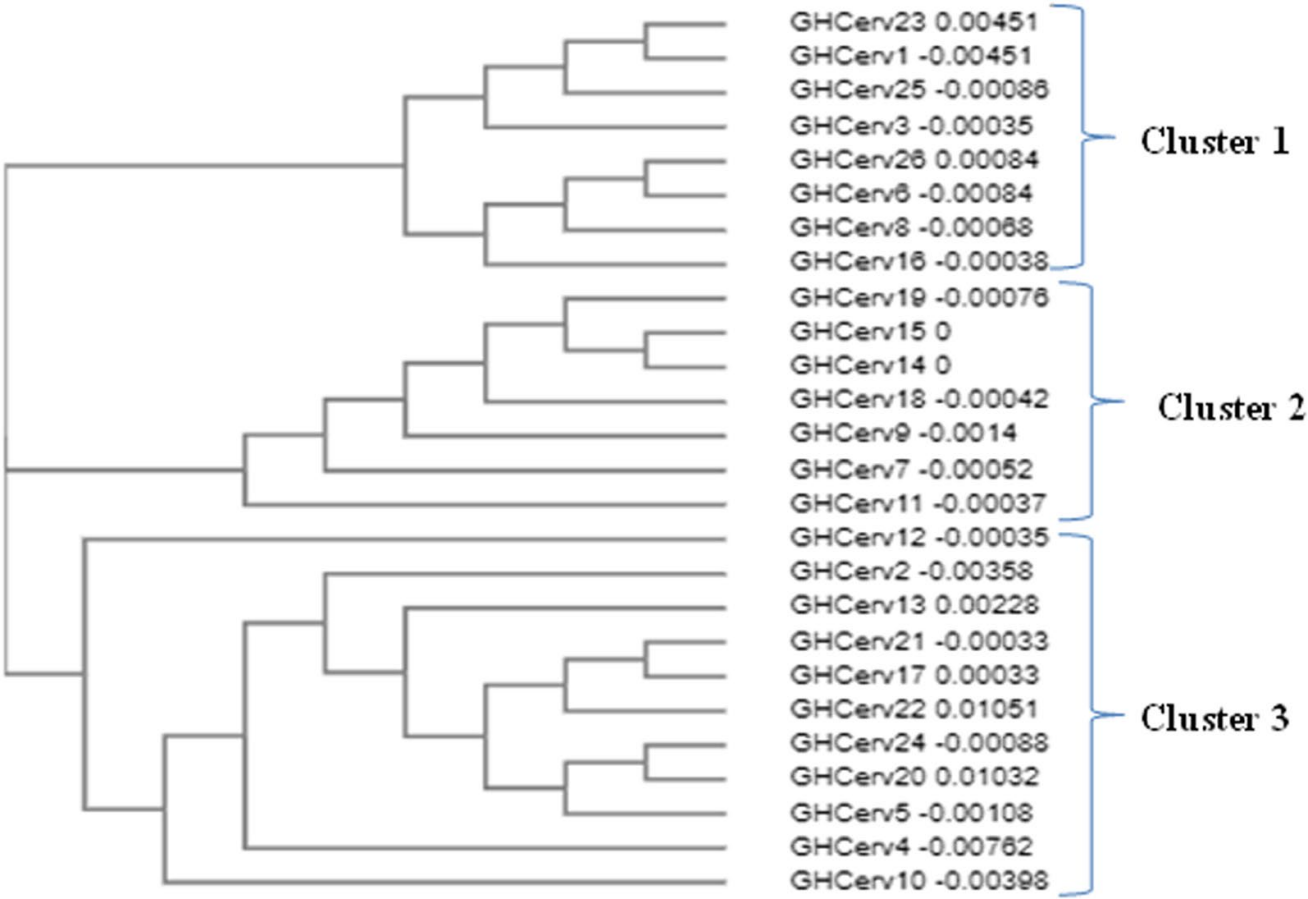
Phylogenetic tree showing HPV 18 L1 sequence similarity in cervical cancers Sequences appeared more similar, clustering into 3 distinct groups

**Fig. 5:**
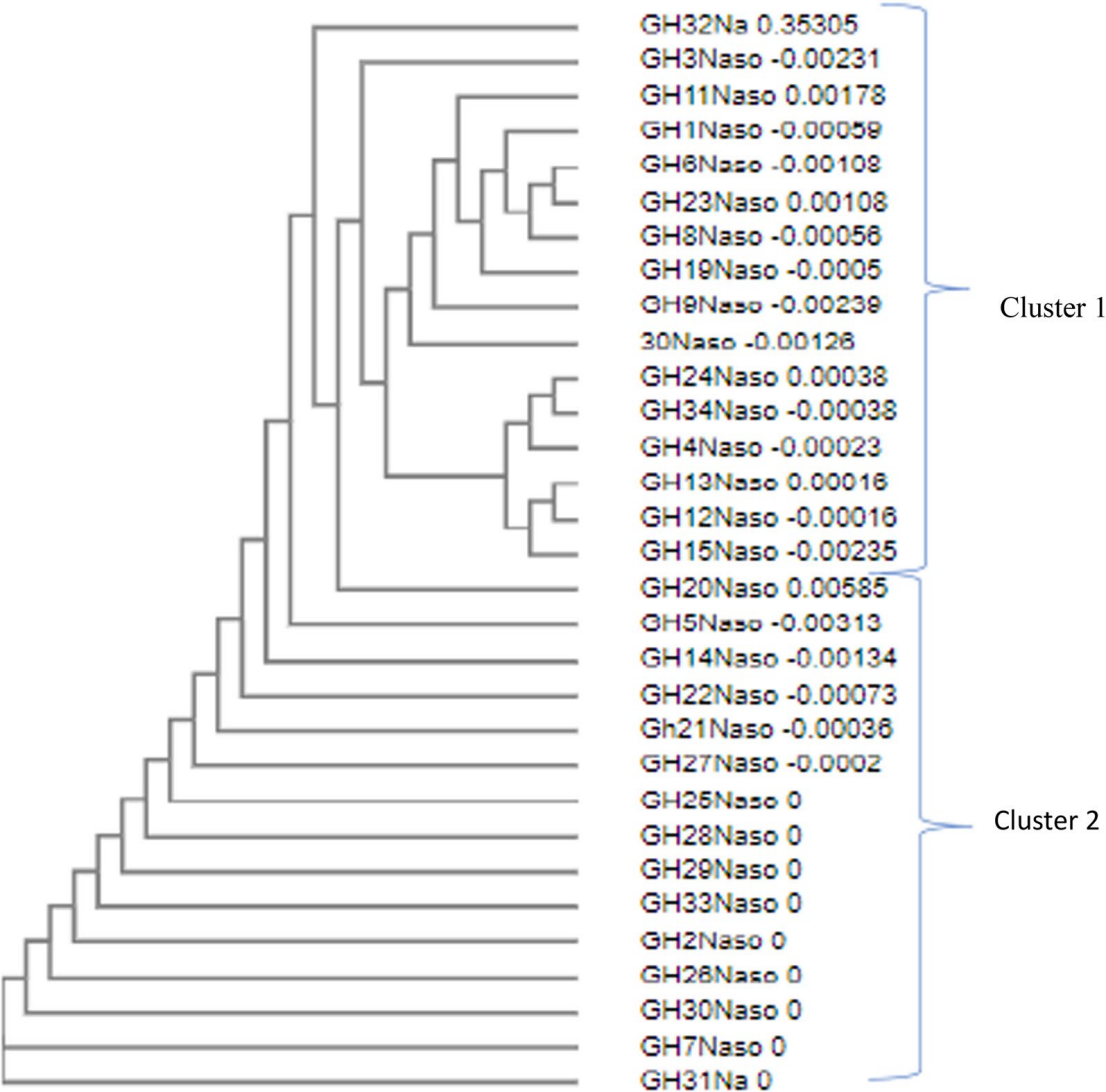
Phylogenetic tree showing HPV 18 L1 sequence similarity in nasopharyngeal cancers. Sequences appeared more varied, clustering into 2 groups of very diverse sequences. Sequences in cluster 1 showed some relatedness as compared to cluster 2 which were very distinct with no clustering

**Fig. 6:**
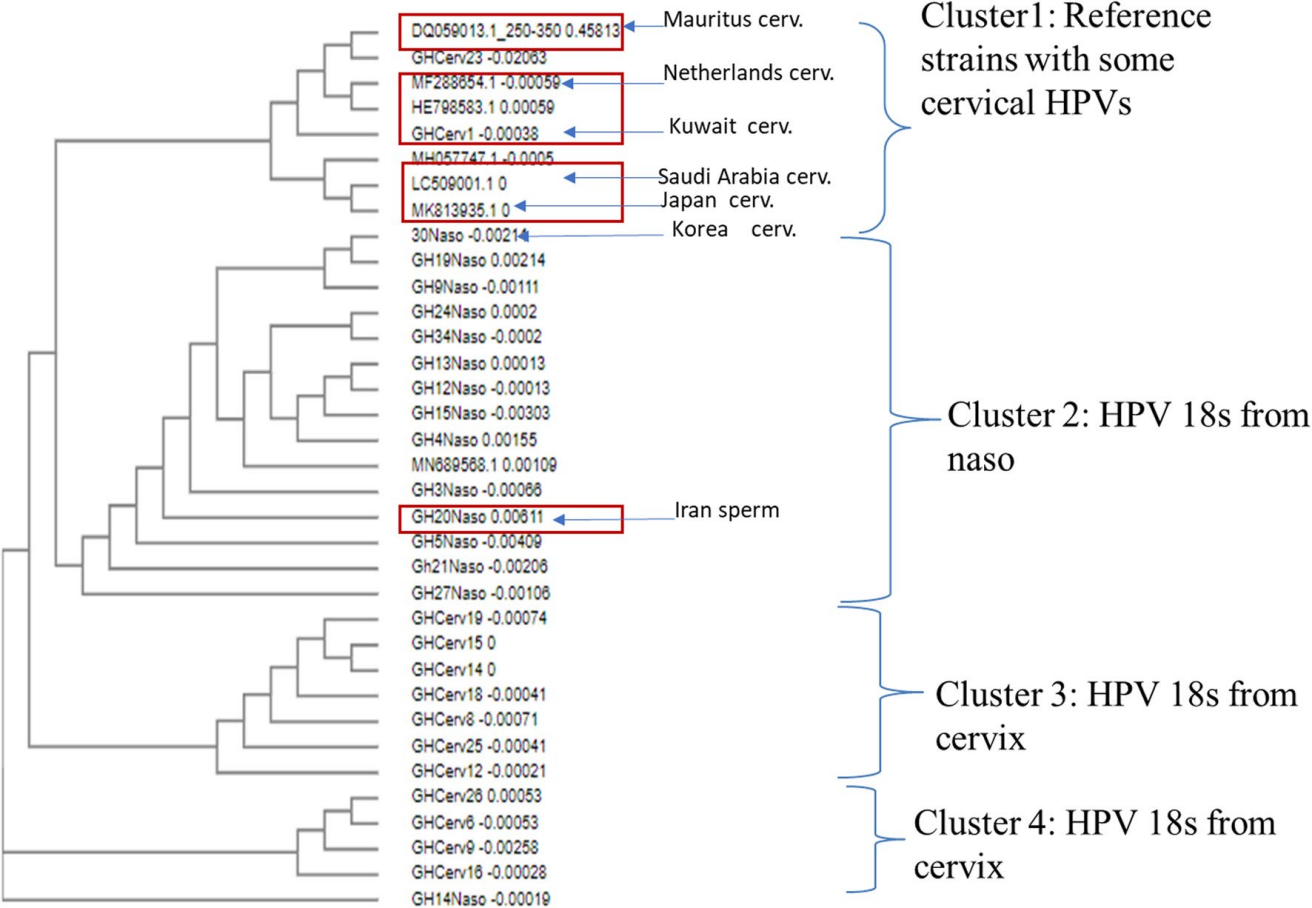
Phylogenetic tree showing HPV 18 L1 sequence from cervical and nasopharyngeal cancers. (13 cervical, 15 nasophrynx). The reference strains from the cervical site (DQ059013-Mauritius, MF288654.1-Netherlands, LC509001.1-Japan, MK813935.1-Korea, MH057747.1-Saudi Arabia) were quite similar and clustered with some of our cervical HPV L1 sequences in cluster 1; the reference strain from sperm (MN689568.1 -Iran) was found in cluster 2 were majority of the L1 sequences from the nasopharynx clustered and cluster 3 and 4 had a majority of the cervical LI sequences

When randomly selected sequences from the nasopharynx and cervix were aligned with reference strains DQ059013 (Mauritius-cervical), MF288654.1 (Netherlands-cervical), LC509001.1(Japan-cervical), MK813935.1 (Korea-cervical), MH057747.1 (Saudi Arabia-cervical) and MN689568.1 (Iran-sperm); the sequences from the cervix were very similar, segregating into 3 main clusters while sequences from the nasopharynx varied with no clearly defined clusters (Figs. 4 and 5). Combined phylogenetic analysis of HPV18 L1 sequences from the cervix, nasopharynx and the reference strains showed sequences clustering into 4 groups namely; clusters 1, 2, 3 and 4 (Fig. 6). Almost all reference strains from the cervix (DQ059013-Mauritius, MF288654.1-Netherlands, LC509001.1-Japan, MK813935.1-Korea and MH057747.1-Saudi Arabia) clustered in cluster 1with a few of our L1 sequences from the cervix. The L1 sequences from the nasopharynx clustered in cluster 2 with the reference strain from sperm (MN689568- Iran) and the rest of the L1 sequences from the cervix clustered in clusters 3 and 4. The L1 sequences from the cervix were very distinct from the sequences from the nasopharynx and there was no clustering between the sequences from the two sites (Fig. 6).

### Sequences and phylogenetic analysis of HPV18 LCR

**Fig. 7:**
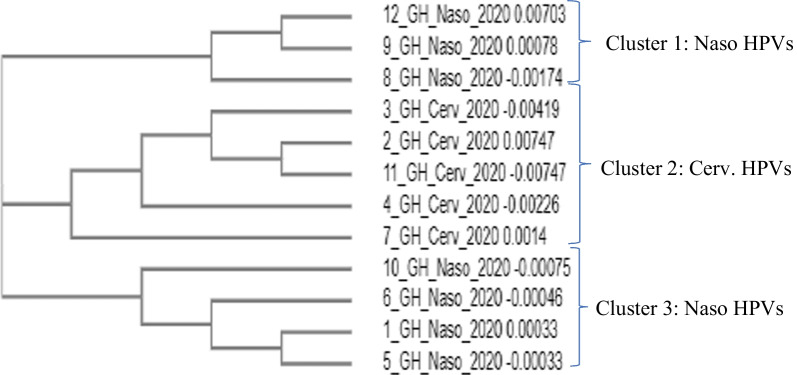
Phylogenetic tree for HPV18 LCR sequences from the nasopharynx (naso) and cervix (cerv). Nasopharyngeal HPVs were more diverse compared to the cervical HPVs which clustered together

A total of 6 each of HPV 18 LCR sequences from the cervical and nasopharyngeal regions were analyzed as in "Sequence and phylogenetic analysis of HPV18 L1 genes" section above. The HPVs clustered by site, with 5 of the cervical HPVs clustering together and one isolated. The nasopharyngeal HPVs however showed more diversity, clustering in two groups of 3 and 2 with one isolated (Fig. 7).

### Differential expression of HPV 18 E6 and E7 oncogenes in cervical and nasopharyngeal cancer tissues

**Fig. 8:**
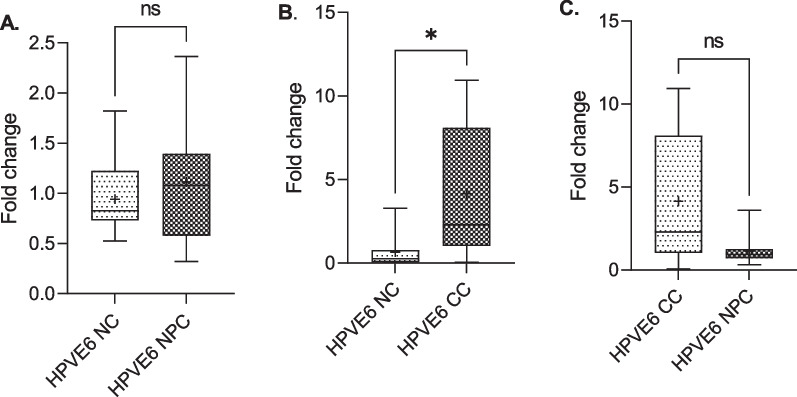
Differential expression of HPV 18 E6 oncogenes in cervical and nasopharyngeal cancer tissues. Higher fold change in HPV18 E6 expression in nasopharyngeal cancer tissues compared to the controls (**A**), higher fold change in HPV18 E6 expression in cervical cancer tissues compared to the controls (**B**), and higher fold change in HPV18 E6 expression in cervical cancer compared to the nasopharyngeal cancers (**C**). NC: negative control, NPC: nasopharyngeal cancer, CC: cervical cancer

**Fig. 9:**
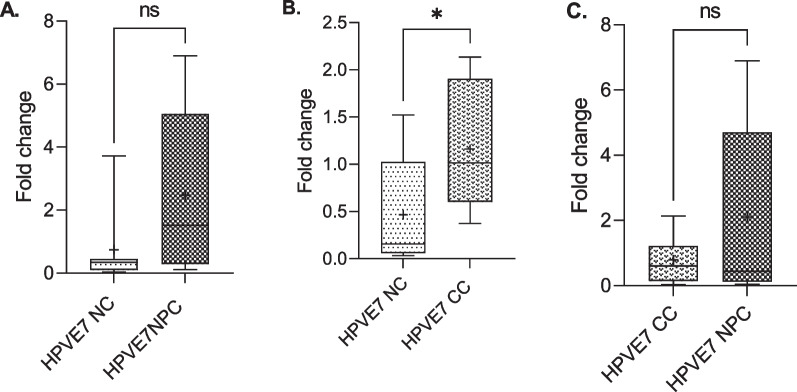
Differential expression of HPV 18 E6 oncogenes in cervical and nasopharyngeal cancer tissues. Higher fold change in HPV18 E7 expression in nasopharyngeal cancer tissues compared to the controls (**A**), higher fold change in HPV18 E7 expression in cervical cancer tissues compared to the controls (**B**) and higher fold change in HPV18 E7 expression in nasopharyngeal cancer tissues compared to the cervical cancer tissues (**C**). NC: negative control, NPC: nasopharyngeal cancer, CC: cervical cancer

The RT-qPCR analysis of 10 RNA samples duplicated each for cervical and nasopharyngeal cancer tissues for HPV 18 E6 and E7 mRNAs expression is presented (Figs. 8 and 9). The results showed a higher expression of E6 in the nasopharyngeal cancer tissues compared to the non-cancer nasopharyngeal tissues, though the difference was not statistically significant (*p* = 0.083), however, the expression was significantly higher in cervical cancer compared to the non-cervical cancer tissues (*p* = 0.035). Comparing both cancer tissues, expression was higher in cervical than nasopharyngeal though not statistically significant (*p* = 0.069; Fig. 8). Similarly, there was a higher expression of HPV18 E7 in the nasopharyngeal cancer tissues compared to the controls, though not statistically significant (*p* > 0.05). HPV18 E7 expression was significantly elevated in the cervical cancer tissues than the controls (*p* < 0.05). Expression of E6 in the cervical cancer tissues was higher than the nasopharyngeal cancer tissues though the difference was not statistically significant (*p* > 0.05; Fig. 8). However, in the nasopharyngeal cancer tissues, E7 was highly expressed compared to that of the cervical cancer tissues (*p* > 0.05; Fig. 9).

## Discussion

The current study describes for the first time the HPV18 L1 and LCR diversity and the possible impact of the genetic drift on transcription pattern of viral oncogenes at cervical and nasopharyngeal sites. Our objective was to determine the distribution of HPVs, analyze the site-specific diversity of L1 gene and LCR nucleotide sequences, and E6 and E7 expression pattern of the prevalent HPV type in cervical and nasopharyngeal cancers tissues.

From our HPV L1 sequences analysis, HPV18 emerged as the predominant HPV type in the two cancers. This is consistent with literature as HPV16 and 18 have been reported as the most common HPV types in cancers [28–32]. Though HPV16 is mostly reported as the predominant HPV type of the two, a study by Donkoh et al. in Kumasi, a city in Ghana, did not detect HPV16 as the most prevalent HPV type while HPV18 was reported as the fourth most common genotype after HPV 52, 56, and 35 among women who had never experienced cervical screening [29]. Generally, the distribution of HPVs is largely known to vary based on geographical location, these geographical differences could explain why HPV18 was the prevalent HPV type observed in our samples, which were mostly obtained from the greater Accra region of Ghana.

Comparison of the L1 gene and LCR sequences of HPV18s from the cervical and nasopharyngeal sites showed great diversity. Our analysis revealed that the L1 gene sequences of HPV18s from the nasopharyngeal cancers were highly dissimilar in relation to each other (59–100%) and shared some similarity with the reference L1 sequence from sperm. In contrast, the L1 gene sequences from the cervical cancers were similar in relation to each other (91.0–100%). Though majority of the L1 sequences from cervical cancer did not cluster with the reference strains from the cervix, they did cluster together showing relatedness, with only a few clustering with the reference strains from the cervix. Of note, none of the reference strains from the cervix and our L1 sequences from cervical cancer clustered with the sequences from the nasopharynx, suggesting a distinct variation between the sequences from the two sites. Additionally, the L1 sequences from nasopharyngeal cancers clustered with the reference strain from sperm. This suggests that the nasopharyngeal HPV 18 share similarity with HPV18 from sperm pointing to some degree of relatedness.

Also, the LCR sequences of HPV18 from the nasopharyngeal cancers were quite different relative to the LCR from cervical cancers. These findings suggest that HPV18 L1 and LCR genes undergo some level of genetic drift in the nasopharyngeal site compared to the cervical site. These changes could be because HPV18 is well adapted to the cervical environment compared to the nasopharyngeal environment. Global analysis of LCR in HPV 18 has shown that the region is highly polymorphic and the sequence variation may adapt the virus to its environment [33]. Indeed, the link between hr- HPV infections and cervical, and other anogenital cancers has long been established [34]. Hr-HPVs have inhabited the cervical region over time and may be well adapted to this site. Contrastingly, hr-HPVs were not associated with nasopharyngeal cancers until recently when emerging evidence suggested a role in the development of a subset of nasopharyngeal cancers [35]. Thus, HPV18s may not be fully adapted to this relatively newer site, hence the sequence variations. Changes in the L1 gene, which encodes the capsid protein; the immunogenic part of the virus, suggest efforts at immune adaptation. These changes may reduce viral fitness by impairing capsid assembly, thus contributing to viral persistence by evading the host immune response [36]. Viral immune evasion could interfere with immunogenicity, with strong implications for the potential development of diagnostic tests, and therapeutic and preventive vaccines for HPVs in the nasopharyngeal region.

The LCR also regulate the transcription of viral oncogenes including E6 and E7, hence changes in the LCR may have implications for the viral gene expression in the nasopharyngeal site which in turn, may have implications on the severity and outcome of the cancer at this site. Indeed, the cellular environment and site of infection are known to affect viral pathogenicity by modulating viral gene expression [16]. This can be observed in cervical and oropharyngeal cancers where the 5-year age-standardized relative survival rate was 64.2% for HPV-positive cervical carcinomas and 51.2% for HPV-positive oropharyngeal carcinomas [37], depicting differences in HPV associated cancer outcomes in the two sites.

Changes in the LCR prompted further analysis to determine the expression pattern of HPV18 E6/E7 mRNA in the cervical and nasopharyngeal regions. Our findings showed a higher fold change in E6 expression in the cervical cancer tissues relative to the nasopharyngeal cancer tissues and the reverse was the case for E7. This implies that the variations in the LCR of HPV18 observed in the two sites impacts the transcription of viral oncogenes. E6 expression has been strongly implicated in tumorigenesis through the induction of increased proteasome-dependent p53 degradation, leading to deregulated cell cycle arrest, DNA repair, and apoptosis [13, 38]. The HPV E7 proteins however, interact with the retinoblastoma protein pRb, resulting in its enhanced phosphorylation and degradation. The pRb destruction leads to the release of E2F family of transcription factors and the subsequent activation of genes promoting cell proliferation and cancer [13]. Thus, the differential expression of the two oncogenes in the two sites suggests a difference in the mechanisms by which HPV18 induce cancer in cervical and nasopharyngeal tissues, and this could possibly explain the differences in survival rate of HPV infection-associated cervical and nasopharyngeal cancers.

## Conclusion

This report compares for the first time the diversity of HPV18 L1 and LCR sequences, and E6/E7 expression patterns in cervical and nasopharyngeal cancers. HPV18 L1 sequences were highly identical in the cervical cancer group than the nasopharyngeal group with percentage nucleotide identities ranging from 91.0–100% to 59–100%, respectively. The cervical HPV18 L1 sequences clustered more with the reference strains compared to sequences from nasopharyngeal cancer tissues. Also in cervical cancers tissues, HPV E6 was highly expressed compared to E7. Contrastingly, a higher expression of HPV18 E7 was observed in nasopharyngeal cancers than E6. These findings clearly demonstrate HPV18 virulence factor expression dynamics in different epithelial cancers, suggesting tissue site-specific response and adaptation.

### Supplementary Information

**Additional file 1. Table S1.** Percentage Matrix Identity for cervical cancer HPV18 L1 sequences.

**Additional file 2. Table S2.** Percentage Matrix Identity for Nasopharyngeal cancer HPV L1 sequence.

## Data Availability

All data generated or analysed during this study are included in this article and supplementary files.

## Abbreviations

HPV: Human Papillomaviruses
Hr-HPVs: High-risk Human Papillomavirus
CC: Cervical cancer
NPC: Nasopharyngeal cancers
LCR: Long Control Region
pRb: Retinoblastoma protein
ORF: Open reading frame
FFPE: Formalin Fixed Paraffin Embedded
DNA: Deoxyribonucleic acid
RNA: Ribonucleic acid
PCR: Polymerase chain reaction
