# Supplementary material for: HPV18 L1 and long control region sequences variation and E6/E7 differential expression in nasopharyngeal and cervical cancers: a comparative study
Source: Infect Agent Cancer. 2023 Dec 1;18:78. doi: 10.1186/s13027-023-00560-5 (PMC10691078; doi:10.1186/s13027-023-00560-5)
Supplement: Supplementary file 1 — Additional file 1. Table S1. Percentage Matrix Identity for cervical cancer HPV18 L1 sequences. [file 13027_2023_560_MOESM1_ESM.docx]

**Table S1. Percentage Matrix Identity for cervical cancer HPV18 L1 sequences**

|  | 77 Cerv | 25 Cerv | 81 Cerv | 80 Cerv | 72 Cerv | 70 Cerv | 50 Cerv | 35 Cerv | 33 Cerv | 73 Cerv |
| --- | --- | --- | --- | --- | --- | --- | --- | --- | --- | --- |
| 77Cerv | 100 | 99.04 | 97.25 | 99.04 | 96.33 | 96.33 | 99.02 | 99.04 | 99.04 | 99.02 |
| 25Cerv | 99.04 | 100 | 99.06 | 99.04 | 99.04 | 99.04 | 99.02 | 95.41 | 95.41 | 99.02 |
| 81Cerv | 97.25 | 99.06 | 100 | 100 | 98.17 | 98.17 | 100 | 98.11 | 98.11 | 100 |
| 80Cerv | 99.04 | 99.04 | 100 | 100 | 100 | 100 | 100 | 100 | 100 | 100 |
| 72Cerv | 96.33 | 99.04 | 98.17 | 100 | 100 | 100 | 100 | 100 | 100 | 100 |
| 70Cerv | 96.33 | 99.04 | 98.17 | 100 | 100 | 100 | 100 | 100 | 100 | 100 |
| 50Cerv | 99.02 | 99.02 | 100 | 100 | 100 | 100 | 100 | 100 | 100 | 100 |
| 35Cerv | 97.12 | 96.19 | 97.14 | 98.02 | 98.08 | 98.08 | 99.02 | 98.10 | 98.10 | 99.02 |
| 33Cerv | 96.12 | 96.12 | 97.09 | 97.09 | 97.09 | 97.09 | 97.06 | 97.09 | 97.09 | 97.09 |
| 73Cerv | 91.82 | 95.19 | 92.66 | 96.15 | 92.66 | 92.66 | 97.06 | 96.15 | 96.15 | 97.06 |

Similarity between cervical cancer (Cerv) HPV L1 sequences. HPV18 L1 sequence were highly identical in this group, with percentage nucleotide identity ranging from 91.0%-100%.
