# Supplementary material for: HPV18 L1 and long control region sequences variation and E6/E7 differential expression in nasopharyngeal and cervical cancers: a comparative study
Source: Infect Agent Cancer. 2023 Dec 1;18:78. doi: 10.1186/s13027-023-00560-5 (PMC10691078; doi:10.1186/s13027-023-00560-5)
Supplement: Supplementary file 2 — Additional file 2. Table S2. Percentage Matrix Identity for Nasopharyngeal cancer HPV L1 sequence. [file 13027_2023_560_MOESM2_ESM.docx]

**Table S2. Percentage Matrix Identity for Nasopharyngeal cancer HPV L1 sequence**

|  | 43 Naso | 52 Naso | 6 Naso | 64 Naso | 5 Naso | 38 Naso | 69 Naso | 41 Naso | 31 Naso | 28 Naso |
| --- | --- | --- | --- | --- | --- | --- | --- | --- | --- | --- |
| 43 Naso | 100 | 59.41 | 63.54 | 63.37 | 63.64 | 64.29 | 63.92 | 64.29 | 64.58 | 64.95 |
| 52 Naso | 59.41 | 100 | 92.16 | 93.46 | 92.38 | 93.27 | 93.20 | 91.35 | 93.14 | 93.20 |
| 6 Naso | 63.54 | 92.16 | 100 | 98.04 | 98.04 | 99.01 | 99.01 | 99.02 | 99.02 | 99.02 |
| 64 Naso | 63.37 | 93.46 | 98.04 | 100 | 99.04 | 99.03 | 97.12 | 99.02 | 99.03 | 99.01 |
| 5 Naso | 63.64 | 92.38 | 98.04 | 98.10 | 100 | 99.04 | 99.03 | 97.12 | 99.02 | 99.03 |
| 38 Naso | 64.29 | 93.27 | 99.01 | 99.04 | 99.04 | 100 | 100 | 98.06 | 100 | 100 |
| 69 Naso | 63.92 | 93.20 | 99.01 | 99.03 | 99.03 | 100 | 100 | 98.06 | 100 | 100 |
| 41 Naso | 64.29 | 91.35 | 99.02 | 97.12 | 97.12 | 98.06 | 98.06 | 100 | 100 | 99.03 |
| 31 Naso | 64.58 | 93.14 | 99.02 | 99.02 | 99.02 | 100 | 100 | 100 | 100 | 100 |
| 28 Naso | 64.95 | 93.20 | 99.02 | 99.03 | 99.03 | 100 | 100 | 100 | 100 | 100 |

Sequence similarity between nasopharyngeal cancer (NASO) HPV L1 sequences. HPV18 L1 sequence were very varied in this group, with percentage nucleotide identities ranging from as low as 59% - 100
